# Enhancement of Pleasure during Spontaneous Dance

**DOI:** 10.3389/fnhum.2017.00572

**Published:** 2017-11-29

**Authors:** Nicolò F. Bernardi, Antoine Bellemare-Pepin, Isabelle Peretz

**Affiliations:** ^1^International Laboratory for Brain, Music and Sound Research (BRAMS), Montreal, QC, Canada; ^2^Department of Psychology, McGill University, Montreal, QC, Canada; ^3^Department of Psychology, University of Montreal, Montreal, QC, Canada; ^4^Centre for Research on Brain, Language and Music (CRBLM), Montreal, QC, Canada

**Keywords:** dance, music, emotions, valence, arousal, movement, kinematics, heart rate variability

## Abstract

Dancing emphasizes the motor expression of emotional experiences. The bodily expression of emotions can modulate the subjective experience of emotions, as when adopting emotion-specific postures and faces. Thus, dancing potentially offers a ground for emotional coping through emotional enhancement and regulation. Here we investigated the emotional responses to music in individuals without any prior dance training while they either freely danced or refrained from movement. Participants were also tested while imitating their own dance movements but in the absence of music as a control condition. Emotional ratings and cardio-respiratory measures were collected following each condition. Dance movements were recorded using motion capture. We found that emotional valence was increased specifically during spontaneous dance of groovy excerpts, compared to both still listening and motor imitation. Furthermore, parasympathetic-related heart rate variability (HRV) increased during dance compared to motor imitation. Nevertheless, subjective and physiological arousal increased during movement production, regardless of whether participants were dancing or imitating. Significant correlations were found between inter-individual differences in the emotions experienced during dance and whole-body acceleration profiles. The combination of movement and music during dance results in a distinct state characterized by acutely heightened pleasure, which is of potential interest for the use of dance in therapeutic settings.

## Introduction

**Figure 1:**
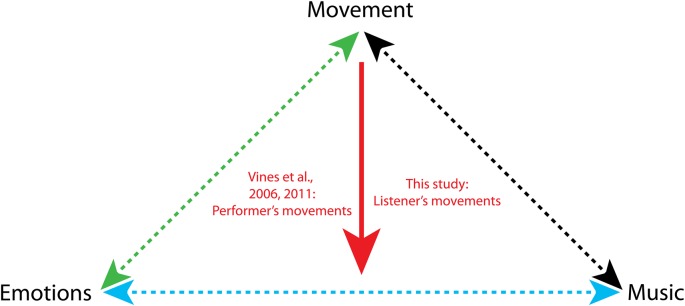
Relationship between movements, music and emotions. The green arrow indicates the bidirectional relationship between emotions and movements (Darwin, 1872; Ekman et al., 1969; Izard, 1971; Meijer, 1989; Stepper and Strack, 1993; Dittrich et al., 1996; Wallbott, 1998; Montepare et al., 1999; Pollick et al., 2001; Sawada et al., 2003; Atkinson et al., 2004, 2007; Niedenthal, 2007; Roether et al., 2009; Carney et al., 2010; Havas et al., 2010; Dael et al., 2012; Havas and Matheson, 2013; Arminjon et al., 2015; de Gelder et al., 2015). The cyan arrow indicates the bidirectional relationship between emotions and music (Shatin, 1970; Fisher and Greenberg, 1972; McFarland, 1985; Nyklíček et al., 1997; Peretz et al., 1998; Blood and Zatorre, 2001; Gomez and Danuser, 2004; Bigand et al., 2005; Baumgartner et al., 2006; Zentner et al., 2008; Coutinho and Cangelosi, 2011; Saarikallio, 2011; Thoma et al., 2012; Koelsch, 2014; Juslin et al., 2015; Vuilleumier and Trost, 2015). The black arrow indicates the bidirectional relationship between movement and music (Clynes, 1977; Flohr and Brown, 1979; Needler and Baer, 1982; Sims, 1985; Davidson, 1993; Worden, 1998; Boone and Cunningham, 2001; Leman, 2007, 2010; Toiviainen et al., 2010; Janata et al., 2012; Burger et al., 2013, 2014; Maes and Leman, 2013; Van Dyck et al., 2013; Witek et al., 2014, 2017; Maes, 2016).

Movements are intimately linked to emotional experiences (Figure 1, green arrow). Movement is the vehicle to express emotions to others, by means of facial, vocal and limb displays. The relationship between emotions and movements is bidirectional: facial and limb movements may prime, bias or enhance certain emotional experiences (Niedenthal, 2007). In this study, we investigate whether the presence of movement while listening to music may modulate the emotional response to music (Figure 1, red arrow).

### Music and Emotions

Music is widely regarded for its capacity to elicit emotions (Figure 1, cyan arrow). Emotion elicitation happens as a result of the appraisal of the music novelty and intrinsic pleasantness (Scherer, 2005), association with autobiographical memories (Jäncke, 2008) and empathy for the person, real or imagined, who is expressing through the music (Vines et al., 2011; Miu and Balteş, 2012; for a review, see Scherer and Coutinho, 2013). Various properties of music sounds have been linked to changes in emotional states. Mode, melodic contour and harmonic complexity have been shown to influence whether a piece of music is perceived as pleasant or unpleasant, whereas tempo, accentuation and rhythmic articulation play a key role in determining the degree of emotional arousal (Schubert, 2004; Gomez and Danuser, 2007). The rhythmic features of music may promote entrainment phenomena at the physiological as well as biomechanical level (Coutinho and Cangelosi, 2011), to the point of creating an urge to move in response to certain kinds of music (Janata et al., 2012). The relationship between music and emotions is bidirectional: being in a specific emotional state has been shown to lead individuals to seek out particular types of music, suggesting a use of music for emotional self-regulation (Saarikallio, 2011; Thoma et al., 2012).

### Music and Movement

Music has also a tight connection to movements (Figure 1, black arrow). Convergent auditory, autonomic and motor pathways in the reticular formation of the brainstem provide a foundation for associating sounds, motor and visceral responses, in a fast and reflex-oriented fashion (Cant and Benson, 2003). Bidirectional auditory-motor connections are also present in various regions of the brain, including the basal ganglia, the planum temporale and the dorso-ventral premotor cortex (Griffiths and Warren, 2002; Grahn and Brett, 2007; Lahav et al., 2007). The density of the connections between auditory and motor centers has made some argue that certain musical phenomena, such as rhythm, are better understood as intrinsically auditory-motor, rather than purely auditory (e.g., Zatorre et al., 2007). Furthermore, music may also involve a visual component, when watching the movements of a musician or a dancer. Watching a performer activates the observer’s motor network, presumably because the human brain understands action partly through motor simulation (Calvo-Merino et al., 2004).

Various studies have shown how music influences movements. In these prior studies, participants without dance training were asked to dance freely to music (Burger et al., 2013; Saarikallio et al., 2013). The music was chosen for expressing or inducing different emotions, such as happiness, anger, sadness or tenderness. These different musical emotions were expressed by distinct dance moves. The movements were generally faster and showed greater spread-out posture of the hands when dancing to happy music compared to sad music (Saarikallio et al., 2013).

### Motor Modulation of the Emotional Response to Music

Prior studies have focused on the relationships between either movement and emotions, or music and movements, or music and emotions (Figure 1, dotted arrows). Surprisingly little is known about the mediation effects that one of these three elements may exert on the relationship between the other two. In the present study, we ask whether the presence or absence of movement modulates the emotional response to music. The existence of such a mediation effect is expected in light of the two-way connections between music, emotions and movements described above.

The only prior study (Vines et al., 2006, 2011) that has investigated the effect of movements on musical emotions, showed that the gestures of the performer influence the emotional experience of the audience. In that study, participants made continuous judgments of emotional tension while watching, listening to, or both watching and listening to pre-recorded music performances. The visual information pertaining to the performer’s movement either increased or reduced emotional tension, depending on the specific passage in the music piece, even in the absence of sound (Vines et al., 2006, 2011; see also Thompson and Russo, 2007).

No study has investigated so far how the movements made *by* *the audience* influence their own emotional experience (Figure 1, red arrow). In other words, the listener’s concurrent body movements have never been taken into account. Understanding whether and under which conditions moving to music may modulate one’s emotional responses may be key to the effective design of psychotherapeutic interventions in which dance is used to sustain emotion regulation (Lesté and Rust, 1990), and to increase motivation in rehabilitation program aimed at restoring motor functions (Hashimoto et al., 2015).

Dance can be broadly defined as the movement of one or more bodies in a choreographed or improvised manner with or without accompanying sound (Bläsing et al., 2012). An enhancement of the emotional response to music during dance may occur by means of two complementary mechanisms. One involves an increased awareness of the emotions expressed by the music and experienced by the listener. Dancing requires attending to the musical stimulus, in order to achieve some degree of congruency or synchronization between the music and the movements. The dancer also needs to identify the emotion state that he or she wants to express, and choose the motor patterns to convey that emotion appropriately. As a result of this process, the emotional state may be brought to a higher level of conscious examination. Consistently with the idea that dance may demand for a certain degree of emotional awareness, it has been shown that individuals with greater dance experience were better at identifying some classes of emotions from facial expressions (Bonny et al., 2017). A second mechanism involves a direct enhancement of the emotional experience due to peripheral stimulation, independently of cognition. Dancing engages the motor system and generates multiple feedback loops, for example in terms of somatosensory, proprioceptive and vestibular stimulation, and physiological arousal. As a result of this increased engagement at multiple levels, the physiological responses underlying emotions are likely to be enhanced when dancing, as well as more likely to reach consciousness (Grandjean et al., 2008), compared to when one listens to music without engaging in a concurrent expressive motor activity. The enhancement of emotional responses when engaging one’s body with the music is also consistent with the framework of embodied cognition (Leman, 2007). Within this framework, it has been suggest that “it is through the body that musical structure is experienced” (Leman, 2010, pp.46), suggesting that a greater engagement of one’s body will facilitate the experience of the musical emotional content.

### Study Aims

The first aim of this study is to investigate whether moving to music modulates music-evoked emotions. We predict that music-evoked emotions would be enhanced when participants generate spontaneous dance movements, as compared to music listening in the absence of movement. Second, we ask whether different facets of the emotional experience, such as valence and arousal, could be differently modulated by dance. We predict an increase of emotional valence, that is the degree of pleasure experienced, during dance, compared to listening in the absence of movements. Furthermore, as a result of the metabolic demands caused by the active production of body movements, we anticipate that dancing to music will be experienced as more arousing, compared to simply listening, particularly when dancing to fast and groovy music. Third, we asked whether the intensity of the emotional experience during dance would correlate with specific elements of the motion pattern. Such a relationship is expected in light of the previous studies, summarized above, which showed that different emotions are represented through distinct dance patterns. For example, music that is perceived as strongly arousing prompts greater acceleration of the head, hands and feet, compared to more relaxing music (Burger et al., 2013). Here, rather than investigating whether music pieces with different features result in different dance patterns, we asked whether people exhibiting different dance patterns would experience emotions in a different way, given the same music excerpts. We expected that individuals who exhibited stronger acceleration patterns would experience stronger emotions, compared to participants with comparatively weaker acceleration patterns.

Merely generating movement sequences, in the absence of music, may increase physiological arousal. In order to examine the role of movements *per se* on emotional experience, in the present study we compared dancing and listening to a third condition that involves the generation of motion patterns closely matched to those generated during dancing, but in the absence of music.

**Figure 2:**
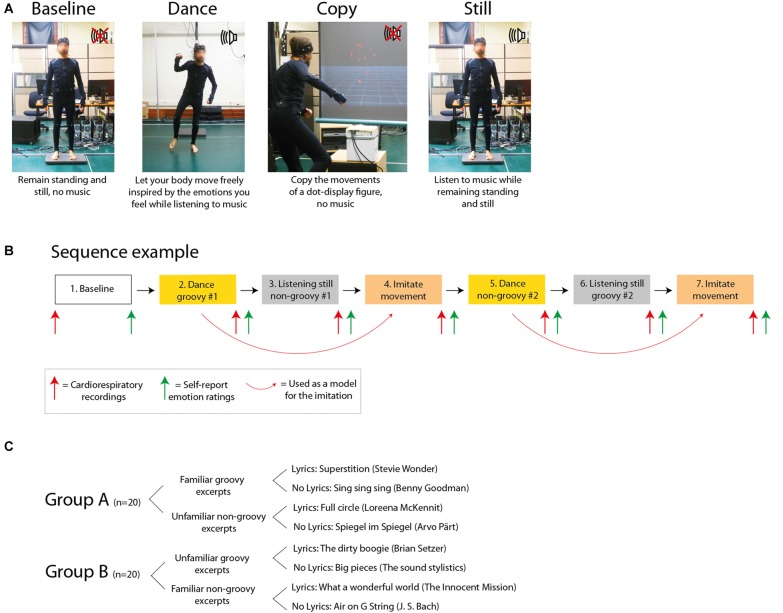
Experimental design. **(A)** Experimental tasks. **(B)** Example of sequence for the experimental tasks. **(C)** Details of the music excerpts.

In sum, we collected emotion ratings, respiration, heart rate and motion capture from participants as they engaged in dancing to music vs. listening to music while remaining still. As additional control conditions, emotional responses from the same participants were also collected following rest and after copying the movements of a dot-display animated human figure, in the absence of music. Unknown to participants, this figure reproduced the exact motion patterns generated by the participants themselves during the dance they had performed shortly before. Overall, the study assessed the impact of the presence vs. absence of both music and movements, in the four possible combinations (Figure 2).

## Materials and Methods

### Participants

We tested 40 participants without motor, cardiovascular or respiratory medical conditions. Participants (19 female) were recruited from the local universities through online boards, and were for the most part university students (age, M ± SD: 26.2 ± 4.9; weight: 64.5 ± 11.1 Kg; height: 170.1 ± 10.1 cm). In terms of education, five were completing high school, 19 had or were completing a bachelor degree, 13 had or were completing a master degree, three had or were completing a doctorate. Hand dominance was right for 38 participants and left for two participants. In terms of ethnicity, 25 were white, 11 were Asian, 3 were Hispanic and 1 was black. Participants reported having slept 7.6 ± 1.4 h on the night prior to the experiment. None of the participants reported dancing professionally, although 11 of them reported dancing as amateurs (average hours dancing/week: 0.7 ± 1.7). All participants had less than 3 years of formal dance training (average years of training: 0.2 ± 0.5). Two participants reported playing a musical instrument professionally, and 14 reported playing as amateurs (average hours playing/week: 1.5 ± 2.6; average years of training: 3.3 ± 4.5). This study was carried out in accordance with the ethics guidelines provided by the Human Research Ethics Committee of Univesité de Montréal (Certification number: CERAS-206-17-002-D) with written informed consent from all subjects, in accordance with the Declaration of Helsinki.

### Design and Task

Figure 2A depicts the design of the experiment, and an example of experimental sequence is shown in Figure 2B. Participants were tested in a single session lasting 2.5 h as follows: (a) standing/resting baseline with the explicit request of avoiding movements (no music and no movements), for 4 min and 15 s, which is the same duration as the music excerpts; (b) dancing to a groovy excerpt (see “Music Excerpts” section; both music and movements present); (c) listening to a non-groovy excerpt while standing and refraining from movements (music present but no movements); (d) copying the movement produced during (b) (movements present but no music); (e) dancing to a non-groovy excerpt; (f) listening to a groovy excerpt while standing and refraining from movements; and (g) copying the movement produced during (e). The order of the conditions b-g was pseudo-randomized across participants. None of the participants reported any fatigue at the end of the session.

Participants listened to pre-recorded instructions before each condition. These recorded instructions included as a background the relevant music excerpt, so that participants could prepare themselves to the mood and style of the music to come. For the dancing condition, participants were instructed to listen to the music, to concentrate on their emotional state and to let their body spontaneously move, as inspired by their feelings. Participants were allowed to move the way they wanted within an area delimited by tape on the floor (area size: 2.8 × 3.3 m). For the listening still condition, participants were instructed to listen to the music, concentrate on their emotional state and remain standing, relaxed and perfectly still. We emphasized to participants to keep their body relaxed, without creating muscle tension. The imitation condition was designed with the goal of matching the movements generated during the dance condition. We sought to eliminate the “dance” component by: (1) replacing the music with a non-musical auditory stimulation (a low-passed white noise sound); and (2) replacing the dance with the imitation of somebody else’s movements. Participants were shown on a projection screen (size: 1.2 × 1.2 m) the dot-display motion capture recording of their own dance. Participants were not told that they would be watching their own performance; many reported that they recognized themselves. The video clip had been preliminarily centered, zoomed and rotated such that the animated figure was visible all the times and was facing the participant most of the time. Participants were instructed to imitate the movements of the animated figure.

The music excerpts, as well as the white noise, were edited so that each lasted exactly 4 min and 15 s. The sound was played by a couple of loudspeakers at a peak sound level of 85 dB. All conditions were followed by a 2-min recording of cardiorespiratory variables (see red arrows in Figure 2B), during which participants were asked to remain still in a relaxed standing posture. An exception to this was condition a), for which physiological recordings were collected before, rather than after, in order to acquire baseline physiological readings prior to any experimental manipulation. During testing for all conditions participants were left alone in the testing room, and the lights in the room were dimmed. This was done to help participants feel at ease and to encourage their freedom of expression, particularly during the dance. After the end of the cardiorespiratory recordings, participants provided ratings on their emotional experience (see green arrows in Figure 2B).

The 40 participants were randomly assigned to two groups of 20 participants each. Each participant danced to one groovy and to one non-groovy excerpt, and listened still to one different groovy and one non-groovy excerpt. The two groups were tested with different music excerpts. Participants in group A were tested with familiar groovy excerpts and unfamiliar non-groovy excerpts. Symmetrically, participants in group B were tested with unfamiliar groovy excerpts and familiar non-groovy excerpts (Figure 2C).

### Music Excerpts

Eight music excerpts were selected for the purpose of this study, deriving from the combination of high/low groove × high/low familiarity × with/without lyrics. Not all music is the same in terms of the motion and emotions it evokes. Particularly relevant for the current investigation, the dimension of *groove* has been described in recent years to express the degree to which a certain piece of music urges the listener to generate movements (Janata et al., 2012; Witek et al., 2014). In this study we aimed to control for the potential effect of groove in mediating emotional responses during dance by including both high-groove and low-groove music excerpts. We also controlled for two potential confounds: familiarity and lyrics. Previous studies showed that both familiarity and the presence of lyrics can play a role in making the listeners emotionally engaged with music (Pereira et al., 2011; Loui et al., 2013). First, we selected two highly familiar excerpts that had been previously validated in the study by Janata et al. (2012) as having a very high degree of groove, one with and one without lyrics. Then, we sought to identify additional music excerpts with high vs. low degree of groove. Following the study of Janata et al. (2012), groove was defined to participants as: “The aspect of music that compels the body to move”. We sought to include excerpts with high and low familiarity, as well as excerpts with and without lyrics. The further six excerpts were selected from an initial pool of 24 excerpts, half with and half without lyrics. We run a pilot on-line survey with 40 participants, which rated on 1–7 Likert scales each excerpt for groove and familiarity (none of the participants in the pilot study participated in the main study). We chose the six excerpts that, together with the two preliminarily selected from Janata et al. (2012), were most different in terms of groove (maximum difference between high-low groove), and most different in terms of familiarity (maximum difference between high-low familiarity). We also sought to minimize the difference in familiarity between the high and low groove excerpts, at both the high and low extremes of the familiarity spectrum. This was done to ensure that we included some excerpts with high groove that were familiar and some that were unfamiliar (and, similarly, some excerpts with low groove that were familiar and some that were unfamiliar). Symmetrically, we tried to minimize the difference in groove between the high and low familiarity excerpts at both the high and low extremes of the groove spectrum. This selection process yielded the pool of eight music excerpts shown in Figure 2C.

**Figure 3:**
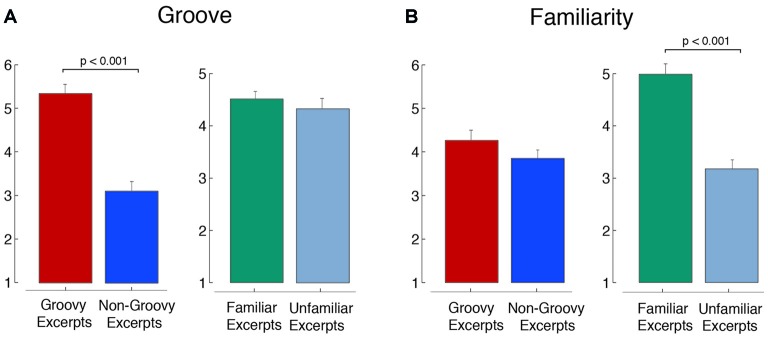
Groove and familiarity in the selected excerpts. The figure shows the ratings of **(A)** groove and **(B)** familiarity for the music excerpts (means ± standard error).

The selected music were found to effectively differentiate between high and low groove and, independently, high and low familiarity. The groovy excerpts were rated by the participants in the main study as having a significantly higher level of groove compared to the non-groovy ones (*t*_(158)_ = 7.79, *p* < 0.001), without this bearing differences on the degree of familiarity (Figure 3A; *t*_(158)_ = 1.23, *p* = 0.22). Symmetrically, the familiar excerpts were rated as significantly more familiar compared to the non-familiar ones (*t*_(158)_ = 6.24, *p* < 0.001), without this bearing differences on the degree of perceived groove (Figure 3B; *t*_(158)_ = 0.58, *p* = 0.56).

### Measures

The primary outcome measure was the emotional rating following each condition. Emotional responses were collected by means of scores provided by the participants on computerized Likert scales. The emotional assessment was based on the dimensional representation of emotions (Russell, 1980), thus using ratings of Valence (1 = very unpleasant, 7 = very pleasant) and Arousal (1 = very relaxing, 7 = very arousing).

To further characterize participants’ level of arousal, in addition to the self-report ratings we recorded physiological arousal in terms respiratory rate, heart rate and heart rate variability (HRV). These dimensions were chosen so as to reflect the state of the autonomic nervous system in response to music listening (Iwanaga et al., 2005). Physiological recordings were made using a wireless portable custom unit, built and designed in house, capable of recording respiratory rhythm, by means of inductive plethysmography using two elastic belts positioned around the abdomen and the chest, and electrocardiogram (ECG), from three standard thoracic leads, at a sampling frequency of 400 Hz (a detailed description of this custom equipment is provided in Codrons et al., 2014). The equipment for the cardiorespiratory recordings was applied to the participant before the beginning of the experiment, and it was worn throughout the experiment. The signals from the inductive plethysmographic belts were analyzed by an interactive program to identify for each breath the positive and negative respiratory peaks and from there the respiratory frequency. The R-R interval was utilized as an indicator for heart rate. First, we obtained the heart period sequence from the ECG. This was done by identifying the peak of the R wave of the ECG, and then constructing the series of the heart period by measuring the R-R interval. This sequence of R-R intervals was converted into a continuous signal at a frequency of 4 Hz by interpolation of the R-R intervals at each data point. The analysis of HRV was performed using the HRVAS toolbox in Matlab (Ramshur, 2010). Frequency-domain HRV was assessed by calculating the power spectrum density in the low frequency (LF, 0.03–0.15 Hz) and high frequency (HF, 0.15–0.6 Hz) bands. The LF band reflects the combined activity of both the parasympathetic and sympathetic branches of the autonomic nervous systems, whereas the HF band is more influenced by parasympathetic modulations (Bernardi et al., 2011). The power within each band was obtained by integrating the power spectrum density between the band frequency limits. For the purpose of statistical analyses, we utilized the percentage of the LF and HF amplitude (LF%, HF%).

Participants’ movements were recorded by means of optical infrared motion capture (Qualysis system) at a frame rate of 100 Hz. We recorded the three-dimensional position of 28 reflective markers attached to each participant, following the marker setup utilized in previous dance experiments and described in details by Burger et al. (2013), comprising (L = left; R = right; F = front; B = back): 1: LF head; 2: RF head; 3: LB head; 4: RB head; 5: L shoulder; 6: R shoulder; 7: breastbone; 8: spine (at the level of the third thoracic vertebrae); 9: LF hip; 10: RF hip; 11: LB hip; 12: RB hip; 13: L elbow; 14: R elbow; 15: L wrist radius; 16: L wrist ulna; 17: R wrist radius; 18: R wrist ulna; 19: L hand; 20: R hand; 21: L knee; 22: R knee; 23: L ankle; 24: R ankle; 25: L heel; 26: R heel; 27: L big toe; 28: R big toe. The analyses were performed using a modified version of the Motion Capture Toolbox (Toiviainen and Burger, 2011). Through a five-step procedure, we sought to compute a limited number of movement variables suggestive of global dance patterns.

First, we reduced the 28 physical markers to a set of 20 secondary markers (Burger et al., 2013), with the aim of reducing redundant information while retaining the essential body parts and joints.

Second, from the three-dimensional joint position data of each secondary marker, we computed several kinematic quantities that have been shown in previous research to discriminate between dance movements in different emotional states (Toiviainen et al., 2010; Burger et al., 2013). Specifically, we computed: the instantaneous velocity (i.e., the first derivative of the marker position), acceleration (i.e., the second derivative of the marker position) and jerk (i.e., the third derivative of the marker position), by means of numerical differentiation; the cumulative distance traveled by the marker; the bounding rectangle, defined as the smallest rectangular area that contains the projection of the trajectory of each marker on the horizontal plane (length of the analysis window: 195 frames); the instantaneous kinetic energy, by means of body-segment modeling (see Toiviainen et al., 2010); the instantaneous distance between the two hands.

Third, the instantaneous values of velocity, acceleration, jerk, kinetic energy and hand distance were averaged over time, for each song (Toiviainen et al., 2010). The time-averaged values for markers that were present on two sides of the body (e.g., right and left hand) were further averaged across the two sides. This resulted in a single score, for each of the kinematic dimensions, for each of the following body landmarks: toe, ankle, knee, sacrum, hip, mid-torso, neck, shoulder, elbow, wrist, hand and head.

Fourth, the dimensionality of the data was reduced by employing Principal Component Analysis (PCA), as implemented in Matlab (version 2015b). The input to the PCA was a matrix containing 80 rows, corresponding to the scores for each participant in the two dance conditions (40 participants × 2 conditions) and 71 columns, corresponding to the kinematic and kinetic variables described above. The data were centered and then factorized using the singular value decomposition algorithm. The analysis was restricted to the first three principal components (PCs), which together explained 96.4% of the total variance. PC1 explained 86% of the variance, and had high positive loadings for features related to jerky and accelerated movements of the feet, ankles, knees, hands, wrists and elbow as well as positive loadings for the cumulative distance of the same body parts. PC1 was therefore interpreted as reflecting fast and widespread whole-body movements, involving a wide exploration of the space in the room (a table with loadings for each PC is provided as Supplementary Material). PC2 explained a further 6.2% of the variance, and had high positive loadings for jerky and accelerated movements of the hands and wrists, while showing negative loadings for the acceleration and jerk of most of the other body parts, the feet in particular. PC2 also presented highly positive loadings for the distance traveled by the hand and wrist markers, and to a lower extent also the other markers. PC2 was therefore interpreted as reflecting a dance pattern predominantly involving fast and expansive movements of the hands, with lower degree of involvement of the lower part of the body. PC3 explained a further 4.2% of the variance, and had high positive loadings for the jerk of upper body parts such as the hands, the wrists, torso, shoulders and head, and negative loads for the acceleration and jerk of the feet and ankles. PC3 also presented negative loadings for the distance traveled by all markers, except the hand. PC3 was therefore interpreted as reflecting a pattern of whole-body dancing “on the spot”, characterized by complex movements of the upper body but minimal exploration of the space.

Finally, we computed a measure of overall movement complexity and a measure of overall movement fluidity, as implemented in the Motion Capture Toolbox (Toiviainen and Burger, 2011). Briefly, the measure of movement complexity is based on the proportion of variance explained by the first five PCs of a PCA run on the whole-body positional data (all joints) from an individual’s motion capture recordings. A low proportion of explained variance implies that the underlying movement is complex, as a high number of PCs is needed to explain the movement sufficiently (see Burger et al., 2013 for further details). Overall movement fluidity was computed as the ratio of velocity to acceleration, from all the available joints. The combination of high velocity and low acceleration reflects fluid movement, whereas the combination of low velocity and high acceleration reflects non-fluid movement.

### Statistics

All the statistical analyses were run using SPSS (version 23). A bivariate two-way repeated measures analysis of variance was run to assess the difference in the emotional response between the various conditions. The dependent variables were the ratings of Valence and Arousal. The independent variables were Movement (2 levels: movement vs. no movement) and Music (2 levels: music vs. no music). Two separate analyses were performed, one for the groovy and one for the non-groovy excerpts. Familiarity and lyrics were not modeled statistically as they did not relate to the hypotheses of the study. The fact that the study design balanced the presence of familiar, unfamiliar, sung and instrumental excerpts reduces the bias that these factors could have on the interpretation of the results. *Post hoc* comparisons were Bonferroni-corrected to take into account the fact that a total of 16 hypotheses were tested (2 movement conditions × 2 music conditions × 2 groove conditions × 2 dependent variables). The alpha level for statistical significance in the *post hoc* tests was therefore set as 0.05/16 = 0.003.

A similar multivariate repeated-measures analysis of variance was run to assess the differences in respiratory rate, heart rate, LF-HRV and HF-HRV between the various experimental conditions. *Post hoc* comparisons were Bonferroni-corrected to take into account the fact that a total of 32 hypotheses were tested (2 movement conditions × 2 music conditions × 2 groove conditions × 4 dependent variables), thus using an alpha level of 0.05/32 = 0.0016.

The relationship between kinematic/kinetic features and the emotion ratings collected following the dance condition was assessed by means of stepwise regression analyses. The independent variables were the five motion capture variables (PC1, PC2, PC3, movement complexity, fluidity). The dependent variable was the rating for valence and, in a separate analysis, the rating of arousal. Groovy and non-groovy excerpts were analyzed together, such that each participant contributed two data points, one for the groovy dance and one for the non-groovy dance. The adjusted R^2^ from the stepwise regression analyses was used as an estimate of the variance explained for each of the dependent variable. The correlations between all dependent measures (emotion ratings, physiological arousal and kinematic) are provided as Supplementary Material.

## Results

### Emotional Valence

**Figure 4:**
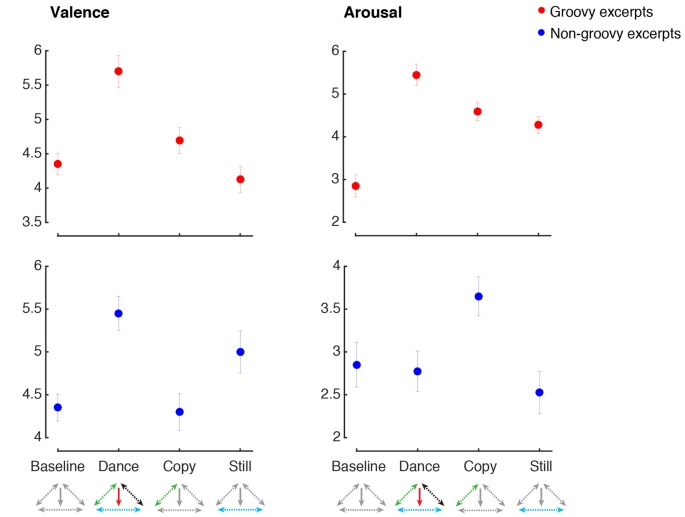
Emotion ratings. The figure shows the emotion ratings for the two emotional dimensions examined, across the four experimental conditions (Baseline, Dance, Copy, Still). Ratings for the groovy excerpts are shown with red markers, and ratings for the non-groovy excerpts are shown with blue markers. Error bars represent means ± standard error. Along the horizontal axis, below the name of the experimental conditions, the model presented in Figure 1 is shown, with colored arrows indicating the component of the model presumably activated in each condition.

Figure 4 shows the mean ratings for self-reported emotional valence and arousal in the baseline, dance, imitation and listening still conditions. When dancing to groovy music, emotional valence was higher during dance compared to all the other conditions (main effect of Music: *F*_(1,38)_ = 3.9, *p* = 0.053, *n.s.*; main effect of Movement: *F*_(1,38)_ = 28.5, *p* < 0.001, $\eta_{\text{p}}^{2}$ = 0.43, power = 0.99; Music by Movement interaction: *F*_(1,38)_ = 10.9, *p* = 0.002, $\eta_{\text{p}}^{2}$ = 0.22, power = 0.97). *Post hoc* comparisons showed that when groovy music was present, participants experienced a higher degree of pleasure if they were engaging in movement, compared to listening still (*p* < 0.001; *p*-values for *post hoc* tests are corrected for multiple comparisons). On the other hand, when participants were making movements, the degree of pleasure was higher if music was present, compared to copying the dance moves in the absence of music (*p* = 0.041). Listening to groovy music in the absence of movement was not regarded as more pleasurable than baseline (*p* > 0.9). Similarly, making groovy-inspired moves in the absence of music was not rated as having higher emotional valence compared to baseline (*p* > 0.9). The pattern was different in the case of non-groovy music. In this case, the degree of pleasure evoked by music was similar when dancing and listening still. Listening to non-groovy music was regarded as more pleasant compared to the conditions without music, regardless for the presence or absence of movement (main effect of Music: *F*_(1,39)_ = 52.4, *p* < 0.001, $\eta_{\text{p}}^{2}$ = 0.57, power = 0.99, *post hoc* test: *p* < 0.001; main effect of Movement: *F*_(1,39)_ = 0.78 *p* = 0.38, *n.s.*; Music by Movement interaction: *F*_(1,39)_ = 1.4, *p* = 0.25, *n.s.*).

### Arousal

Groovy music significantly increased the subjective ratings of arousal, regardless of whether participants were making movements or not (main effect of Music: *F*_(1,38)_ = 24.9, *p* < 0.001, $\eta_{\text{p}}^{2}$ = 0.40, power = 0.99, *post hoc* test: *p* < 0.001; Music by Movement interaction: *F*_(1,38)_ = 1.7, *p* = 0.21, *n.s.*). Engaging in movements inspired by groovy music also resulted in an increase of subjective arousal, regardless of whether the music was actually present (main effect of Movement: *F*_(1,38)_ = 40.9, *p* < 0.001, $\eta_{\text{p}}^{2}$ = 0.52, power = 0.99, *post hoc* test: *p* < 0.001). Contrarily to the groovy excerpt, non-groovy music had the effect of decreasing arousal, compared to the conditions where music was absent (main effect of Music: *F*_(1,39)_ = 10.4, *p* = 0.003, $\eta_{\text{p}}^{2}$ = 0.21, power = 0.88, *post hoc* test: *p* = 0.041). The relaxing effect of non-groovy music was observed regardless for the presence or absence of concurrent movements (Music by Movement interaction: *F*_(1,39)_ = 1.4, *p* = 0.24, *n.s.*). Movements based on non-groovy music did not produce significant changes in the level of arousal (main effect of Movement: *F*_(1,39)_ = 5.2, *p* = 0.029, $\eta_{\text{p}}^{2}$ = 0.12, power = 0.6, *post hoc* test: *p* = 0.46, *n.s.*).

### Physiological Arousal

To quantify participants’ level of arousal beyond subjective ratings, we assessed physiological arousal in terms respiratory rate, heart rate and HRV.

**Figure 5:**
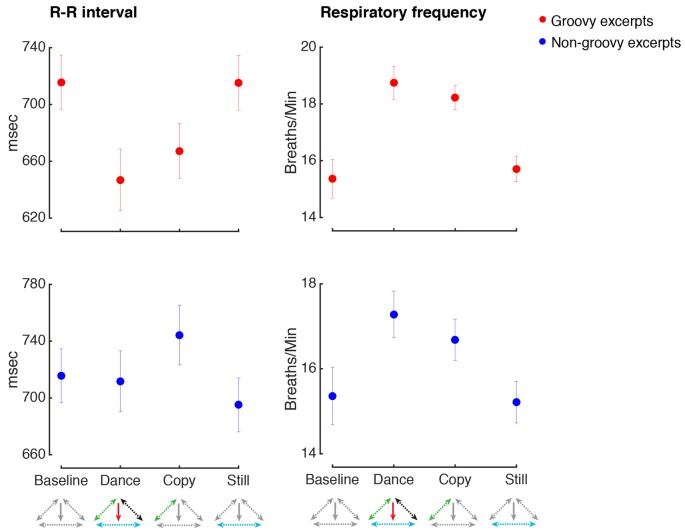
Physiological arousal. All conventions as in Figure 4. The R-R interval indicates the time elapsed between successive heart beats, with higher R-R intervals indicating slower heart rate.

Figure 5 shows the values for respiratory rate and heart rate in the various conditions. Making movements inspired by groovy music increased heart rate and respiratory rate, regardless for the presence or absence of music (heart rate, main effect of Music: *F*_(1,33)_ = 2.3, *p* = 0.14, *n.s.*; main effect of Movement: *F*_(1,33)_ = 18.8, *p* < 0.001, $\eta_{\text{p}}^{2}$ = 0.36, power = 0.99, *post hoc* test: *p* = 0.004; Music by Movement interaction: *F*_(1,33)_ = 1.9, *p* = 0.17, *n.s.*; respiratory rate, main effect of Music: *F*_(1,33)_ = 2.4, *p* = 0.13, *n.s.*; main effect of Movement: *F*_(1,33)_ = 51.3, *p* < 0.001, $\eta_{\text{p}}^{2}$ = 0.61, power = 0.99, *post hoc* test: *p* < 0.001; Music by Movement interaction: *F*_(1,33)_ = 0.2, *p* = 0.64, *n.s.*).

**Figure 6:**
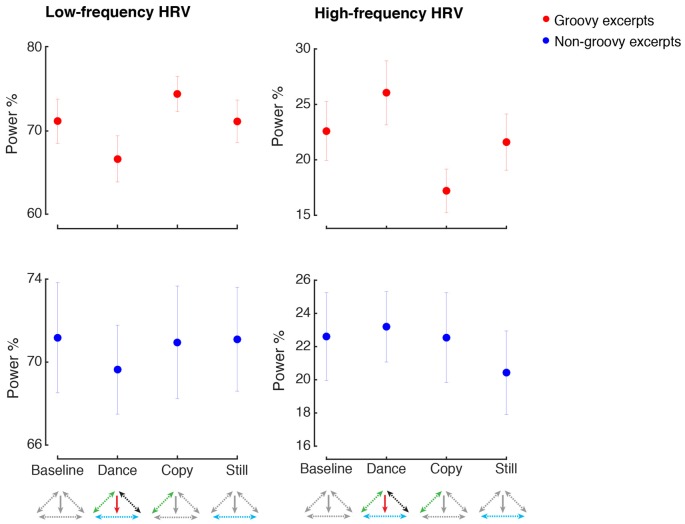
Heart rate variability. All conventions as in Figure 4. HRV, Heart Rate Variability. HRV is expressed as the percentage of the low-frequency (LF) and high-frequency (HF) amplitude.

However, the pattern of physiological responses when making movements with or without music was not identical. As shown in Figure 6, when dancing to groovy music, the parasympathetic-related modulation of heart rate was significantly higher compared to when similar movements were produced in the absence of music (HF HRV, Music by Movement interaction: *F*_(1,33)_ = 5.6, *p* = 0.024, $\eta_{\text{p}}^{2}$ = 0.15, power = 0.81, *post hoc* test: *p* = 0.016). Importantly, the increase in HF HRV during dance compared to imitation was not confounded by concurrent changes in respiratory rate, as the difference in breathing rate between these two conditions was not statistically significant (*p* > 0.9). No other changes in HF or LF HRV were found following the experimental manipulations (all *p* > 0.05).

An increase of physiological arousal was observed when making movements inspired by non-groovy music, regardless of whether music was present or not (respiratory rate, main effect of Movement: *F*_(1,35)_ = 16.6, *p* < 0.001, $\eta_{\text{p}}^{2}$ = 0.32, power = 0.98, *post hoc* test: *p* = 0.008). No other changes in physiological arousal were observed for the manipulations related to non-groovy excerpts (all *p* < 0.05).

### Dance Moves and Emotional Judgments

A small (~4%) portion of the variance in the ratings of emotional valence was explained by overall movement complexity, with more complex movements being associated with more positive valence (*t* = 2.1, *p* = 0.037, Beta = 0.23, Adj R^2^ = 0.043). On the other hand, 60% of the variance in the ratings of emotional arousal was explained by a model including PC1, PC3, movement complexity and fluidity (Adj R^2^ = 0.60). The more participants engaged in fast and widespread whole body movements involving an exploration of the space, as reflected in higher scores of PC1, the more they experienced a sense of arousal (PC1: *t* = 3.6, *p* = 0.001, Beta = 0.48). The other three predictors had an opposite effect, showing that arousal was lower when movements were more complex (*t* = −2.6, *p* = 0.13, Beta = −0.20), more fluid (*t* = −2.8, *p* = 0.006, Beta = −0.37) and when dancing “on the spot” without moving around (PC3, *t* = −2.3, *p* = 0.026, Beta = −0.18).

## Discussion

In this study we recorded the emotional responses of individuals without dance training as they engaged in listening to music, with or without concurrent free dance moves. We showed that dancing to groovy music produced a state of heightened pleasure and arousal. Strikingly, listening to groovy music in the absence of motion was experienced as arousing but not pleasurable, suggesting that a certain degree of embodiment may be necessary to fully enjoy this kind of music. Mechanically imitating the groovy-dance moves, in the absence of music, maintained a state of arousal similar to that observed during dance, but without its associated enhanced pleasure. The findings suggest that dance may amplify the emotional response to a music stimuli beyond what is observed when listening or movement occur in isolation.

According to the embodied cognition framework (Niedenthal et al., 2005; Leman, 2007), the activation of motor patterns that are typically associated with various emotional feelings would contribute to reactivate the broader neural pathways responsible for these emotions. Thus, the emotions triggered when dancing would be more intense, compared to when one listens in stillness, because a more complete emotional representation is reactivated. The music stimuli in our experiment provided a content, a context and a trigger for participants to experience some emotions and to express these through their movements. This presumably explains why movement alone, in the imitation condition, was not sufficient to generate the full pattern of emotional responses. The imitation of movements in the absence of music would not afford an occasion for embodying and experiencing emotions. Instead, imitating movements appeared more akin to a form of physical exercise, relatively neutral in terms of emotional valence, although stimulating in terms of arousal.

The effects of movements and postures on valence and arousal can also be direct, independently of cognitive factors. In a classic study, Stepper and Strack (1993) showed that adopting an upright or slumped posture increased and decreased, respectively, the feeling of pride when receiving the news of an accomplishment (see also Duclos and Laird, 2001; Koch, 2014). Numerous other studies have shown that adopting or inhibiting certain facial expressions changes the way emotional information is processed or remembered (Havas et al., 2010; Havas and Matheson, 2013; Arminjon et al., 2015). Posture-dependent changes in affective state can be found not only in self-reported ratings of emotional intensity, but also in physiological markers such as testosterone increase and cortisol decrease when adopting power poses (Carney et al., 2010). A direct influence of movements on affective response can emerge even in the absence of overt motor execution. Motor imagery or motor observation of actions expressing emotions such as happiness, sadness or fear has been shown to enhance the corresponding affective state in the observer (Shafir et al., 2013). Furthermore, the observation of emotional movements can activate the brain areas involved in emotion processing (Hadjikhani et al., 2009; Kret et al., 2011). This kind of direct effects of physical postures on emotions is likely to contribute to the emotional enhancement described here. In fact, the full-body moves inspired by the dance typically entail a variety of postures associated with emotions such as joy, power and elation.

A further explanation for the emotional enhancement observed during dance calls upon the idea that dancing may enable a fuller appreciation of music, by aiding an embodied perception and more intuitive understanding of the music (Leman, 2010). For instance, body movements can disambiguate meter interpretation of music (Phillips-Silver and Trainor, 2005, 2007). Several structural features of groovy music suggest that its structure stimulates human movements. For example, the relatively fast tempo matches the natural oscillatory frequencies of locomotion and arm-swing (Trainor, 2007), the clear pulse facilitates the entrainment of biomechanical oscillators (Zentner and Eerola, 2010) and the energy in the upbeat provided by syncopation may aid the generation of movements directed against gravity, thus encouraging the continuation of the motion pattern (Fitch, 2016). Underlying this idea is the view that entrainment to the rhythm is a fundamental mechanism for generating emotions in music (Juslin, 2013).

If movements contribute to the emotional experience of music, one should observe correlations between the movements made and the emotions felt. The findings show that motion patterns characterized by a high degree of acceleration and jerk accounted for up to 60% of the variance in the ratings of arousal felt by participants. Although this study does not allow to establish a cause-effect directionality, this finding suggests the existence of a strong relationship between the movements generated when dancing and the feelings of arousal experienced. The pattern described here closely resembles previous findings that showed a positive correlation between hand/feet acceleration and ratings of arousal perceived in the music, as well as a negative correlation between movement fluidity and perceived arousal (Burger et al., 2013). The relationship between movement and felt emotions was more subtle in the case of emotional valence, with kinematic features explaining only a small amount of variance in the valence ratings. With reference to the cognitive and direct routes for emotional enhancement described earlier, we speculate that the increase in arousal could be primarily mediated by direct, body-based mechanisms, grounded in the physiology of the autonomic nervous system, and therefore highly consistent across different participants. Conversely, the increase in emotional valence could be more strongly influenced by cognitive processes such as the degree of emotional awareness, and thus would be more idiosyncratic and depend less on the specific motion patterns produced. Evidence in support of the idea that two different mechanisms may underlie the increase in valence and arousal is provided by the fact that movement complexity was found to influence valence and arousal in opposite ways, with more complex dance patterns being experienced as more pleasant but less arousing.

The changes in emotional responses we observed here can be framed as a case of up-regulation of positive emotions as a result of engaging in dancing (Shafir, 2016). This is seen as an increase in emotional valence during dancing, compared to imitation or listening in stillness, confirmed by the positive correlation between kinematic movement and valence ratings. Emotion regulation is broadly defined as the set of processes whereby people influence the nature, the intensity, the time course and the subjective meaning of their emotions (Gross, 1998). Skilled emotion regulation is believed to significantly impact well-being, including physical health (Sapolsky, 2007), mental health (Gross and Muñoz, 1995), relationship satisfaction (Murray, 2005) and work performance (Diefendorff et al., 2000). As previously described, specific movement patterns, postures and breathing patterns are associated with specific emotions. Engaging in these motor behaviors can enhance the associated emotion, thus offering a chance to upregulate the desired emotions. Emotion regulation during dance may also occur as a result of the physical exercise component. Exercise can reduce depressive and anxious symptoms in both clinical and non-clinical populations (Asmundson et al., 2013; Josefsson et al., 2014). These effects are mediated by both psychological mechanisms, such as increased self-efficacy and distraction from the stressors (Morgan, 1985; Rimer et al., 2012), and physiological mechanisms, such as the release of endogenous opioids in frontolimbic brain circuits following physical exercise (Boecker et al., 2008; see Shafir, 2015 for a review). Similarly, the observation here of an increase in HF HRV during the groovy-dance condition compared to the imitation condition may reflect up-regulation of positive emotions. HRV in the HF range is due to parasympathetic modulation via the vagal nerve, which helps to maintain the dynamic autonomic regulation important for cardiovascular health (Thayer et al., 2012), and successful emotion regulation (Ingjaldsson et al., 2003; Butler et al., 2006). It is noteworthy that this effect was specifically observed when contrasting the dance and the imitation conditions, despite these conditions similarly involved the generation of movements. This finding further corroborates the idea that the combination of music and movement during dance provides an occasion to experience and express positive emotions, resulting in increased pleasure and associated parasympathetic activity.

A limitation to be aware of in the present study pertains to the nature of the imitation condition. In contrast to the dancing, which was done spontaneously and without thinking, copying requires a cognitive effort, which may have on its own reduced the emotional response. The fact that the degree of self-recognition during the imitation was not controlled represents another limitation of this condition. In fact, recognizing one’s moves may have itself generated emotional responses, introducing a source of uncontrolled variability in the ratings of arousal and valence.

In conclusion, we have shown that the emotional response to music is influenced by the concurrent execution of body dance movements. Dancing to groovy music results in substantially increased feelings of pleasure, compared to listening in the absence of motion, even in individuals without previous dance training. These findings also provide an empirical rationale for the use of dance in numerous health-related applications. Dance may offer a safe and engaging context for the development of emotional regulation processes, which represent an important component of most approaches to psychological well being. Dance may also be effectively used in physical rehabilitation, as the up-regulation of positive emotions observed during dance may support motivation and adherence to the practice regimens.

## Author Contributions

NFB and IP designed the experiment; NFB designed data collection and analysis tools; NFB and AB-P collected the data, analyzed the data; NFB, AB-P and IP drafted and revised the article.

## Conflict of Interest Statement

The authors declare that the research was conducted in the absence of any commercial or financial relationships that could be construed as a potential conflict of interest.
